# Nitric oxide mediated inhibition of antigen presentation from DCs to CD4^+^ T cells in cancer and measurement of STAT1 nitration

**DOI:** 10.1038/s41598-017-14970-0

**Published:** 2017-11-13

**Authors:** Joseph Markowitz, Jiang Wang, Zach Vangundy, Jia You, Vedat Yildiz, Lianbo Yu, Isaac P. Foote, Owen E. Branson, Andrew R. Stiff, Taylor R. Brooks, Brandon Biesiadecki, Thomas Olencki, Susheela Tridandapani, Michael A. Freitas, Tracey Papenfuss, Mitch A. Phelps, William E. Carson

**Affiliations:** 1 Moffitt Cancer Center Department of Cutaneous Oncology, Tampa, United States; 2 Department of Oncologic Sciences USF Morsani School of Medicine, Tampa, United States; 3 Comprehensive Cancer Center, The Ohio State University Wexner Medical Center, Columbus, United States; 4 Department of Biomedical Informatics, The Ohio State University Wexner Medical Center, Columbus, United States; 5 Department of Physiology and Cell Biology, The Ohio State University, Columbus, United States; 6 Division of Medical Oncology, The Ohio State University Wexner Medical Center, Columbus, United States; 7 Department of Surgery, The Ohio State University Wexner Medical Center, Columbus, United States

## Abstract

Myeloid derived suppressor cells (MDSC) produce nitric oxide (NO) and inhibit dendritic cell (DC) immune responses in cancer. DCs present cancer cell antigens to CD4^+^ T cells through Jak-STAT signal transduction. In this study, NO donors (SNAP and DETA-NONOate) inhibited DC antigen presentation. As expected, MDSC isolated from peripheral blood mononuclear cells (PBMC) from cancer patients produced high NO levels. We hypothesized that NO producing MDSC in tumor-bearing hosts would inhibit DC antigen presentation. Antigen presentation from DCs to CD4^+^ T cells (T cell receptor transgenic OT-II) was measured via a [^3^H]-thymidine incorporation proliferation assay. MDSC from melanoma tumor models decreased the levels of proliferation more than pancreatic cancer derived MDSC. T cell proliferation was restored when MDSC were treated with inhibitors of inducible nitric oxide synthase (L-NAME and NCX-4016). A NO donor inhibited OT II T cell receptor recognition of OT II specific tetramers, thus serving as a direct measure of NO inhibition of antigen presentation. Our group has previously demonstrated that STAT1 nitration also mediates MDSC inhibitory effects on immune cells. Therefore, a novel liquid chromatography-tandem mass spectrometry assay demonstrated that nitration of the STAT1-Tyr701 occurs in PBMC derived from both pancreatic cancer and melanoma patients.

## Introduction

Melanoma cells are recognized by the immune system, but the anti-tumor activity of T cells and natural killer (NK) cells is inhibited by multiple mechanisms mediated by immune suppressor cells including depletion of nutrients from the tumor microenvironment, production of reactive oxygen and nitrogen species, secretion of immune-suppressive cytokines and induction of additional inhibitory immune cells^1^. Presentation of antigens to T cells by dendritic cells (DCs) is defective in the setting of melanoma^2^. Recently, it has been shown that stimulation of DCs with type I interferons (IFN-α and β) and down-stream signal transduction via the Janus kinase-signal transducer and activator of transcription (Jak-STAT) pathway is critically important to immune surveillance and the generation of effective host T cell immune responses to cancer^3,4^. Furthermore, in dendritic cells, IFN-α signaling is responsible for up-regulation of class I and class II MHC molecules for the presentation of antigens by dendritic cells^5–7^. It has been demonstrated that the anti-tumor effects of IFN-α were dependent on STAT1 signal transduction in immune cells via phosphorylation of tyrosine 701^8^. Jak-STAT signaling was markedly inhibited in human peripheral blood immune cells from tumor bearing patients^9^, More recently, we discovered that the mechanism of immune inhibition involves secretion of NO by tumor-induced inhibitory immune cells (known as myeloid-derived suppressor cells, MDSC) and decreased phospho-STAT1 generation in response to interferon stimulation^10^. Nitration and phosphorylation events have been studied in other proteins as well. In the case of cytochrome c, phosphorylation occurs in both homeostatic and stress processes, whereas nitration only occurs under conditions of stress^11–13^. An analogous process occurs for STAT1 in that phosphorylation of STAT1 is a natural product of interferon signaling and the protein is nitrated in immune cells when exposed to cancer derived myeloid derived suppressor cells^10^.

MDSC arise from myeloid precursors in response to tumor-derived growth factors and pro-inflammatory cytokines (e.g., IL-6, GM-CSF), and their numbers correlate with tumor burden and are predictive of low overall survival. In humans, MDSC are described by their functional ability to suppress T cell activation and their immature myeloid phenotype (typically CD33^+^, CD11b^+^, HLADR^low/−^). In melanoma, it has been shown that MDSC numbers increase in patients with poor response to ipilimumab therapy and levels of NO in MDSC increase with more advanced stages of melanoma^14^. Reports suggest that granulocytic (CD15^+^) or monocytic (CD14^+^) subsets may have unique functional properties^14–16^. Given our discovery that MDSC nitrated STAT1 and the importance of DC Jak-STAT signaling in the generation of an effective host immune response, we hypothesized that MDSC-derived NO would reduce antigen presentation to T cells by dendritic cells.

In order to measure nitrated STAT1, we developed a novel mass spectrometry technique to detect whether elevated levels of nitrated STAT1 would be found in the immune cells of cancer patients. Accurate and sensitive quantification of gene expression is readily accessible to basic scientists and clinical investigators. However, accurate quantification of protein expression and post-translational modifications remains technically challenging. Methods such as immunohistochemistry, immunoblotting, or flow cytometry are extremely useful for identifying qualitative biological changes in disease or response to drug therapy^17^. While flow cytometry can be quantitative for cell-surface proteins or some select intracellular proteins, these methods are largely incapable of determining accurate quantities of intracellular proteins or protein modifications present in biological specimens. Strategies to measure nitration without damaging the protein include: nuclear magnetic resonance, electron paramagnetic resonance, and electronic absorption spectra^18,19^. Proteomic approaches using older mass spectrometry methods capable of high mass accuracy have made significant advancements in characterizing the various protein and protein modification biomarkers present within complex biological samples^20^. However, these methods are not quantitative and therefore do not enable investigators to determine accurate thresholds for differentiating disease vs. normal or dose-response relationships. Recently, specific mass spectrometry methods have been developed to quantitate post-translational modifications, but the application to human clinical samples has been limited by cumbersome labeling techniques such as adding chemical groups to peptides for accurate measurements^21^. We utilized a novel and highly sensitive liquid chromatography-tandem mass spectrometry (LC-MS/MS) method that enables accurate quantification of proteins and their modifications. A selective reaction monitoring (SRM) assay was developed to specifically measure the expression and nitration status of tyrosine-701 (site of phosphorylation that leads to dimerization and subsequent translocation to the nucleus) of the STAT1 protein^22^. This assay was able to accurately quantify both the GTGYIK and GTGnYIK peptides, representing STAT1 and nitrated STAT1, respectively, in laboratory and clinical samples after digestion with trypsin.

Recently, it has been shown that increases in MDSC producing NO as detected by increases in iNOS levels or intracellular NO stain (DAF-FM) are associated with worse prognosis in patients treated with either anti-CTLA-4 or anti-PD-1 antibodies^14,23^. Given the importance of STAT1 in antigen presentation, we hypothesized that NO inhibits antigen presentation by DCs to CD4+ T cells. Low levels of nitric donors were utilized for the antigen presentation experiments. At concentrations 20 fold higher than utilized for the antigen presentation experiments, SNAP may generate free radicals and alter cellular microtubule organization^24^. In dog models, high concentrations of SNAP led to free radical formation and resulted in myocardial damage after ischemic reperfusion^25^. We explored this hypothesis first in a murine model in which homozygous transgenic mice expressing the mouse alpha-chain and beta-chain T cell receptor that pairs with the CD4 co-receptor and is specific for chicken ovalbumin 323–339 in the context of class I-A b. This model is designed for antigen presentation experiments between DC and CD4^+^ T cells and showed that NO inhibits antigen presentation from DC to CD4^+^ T cells.

## Results

### NO decreases antigen presentation from DC to CD4^+^ T cells

**Figure 1:**
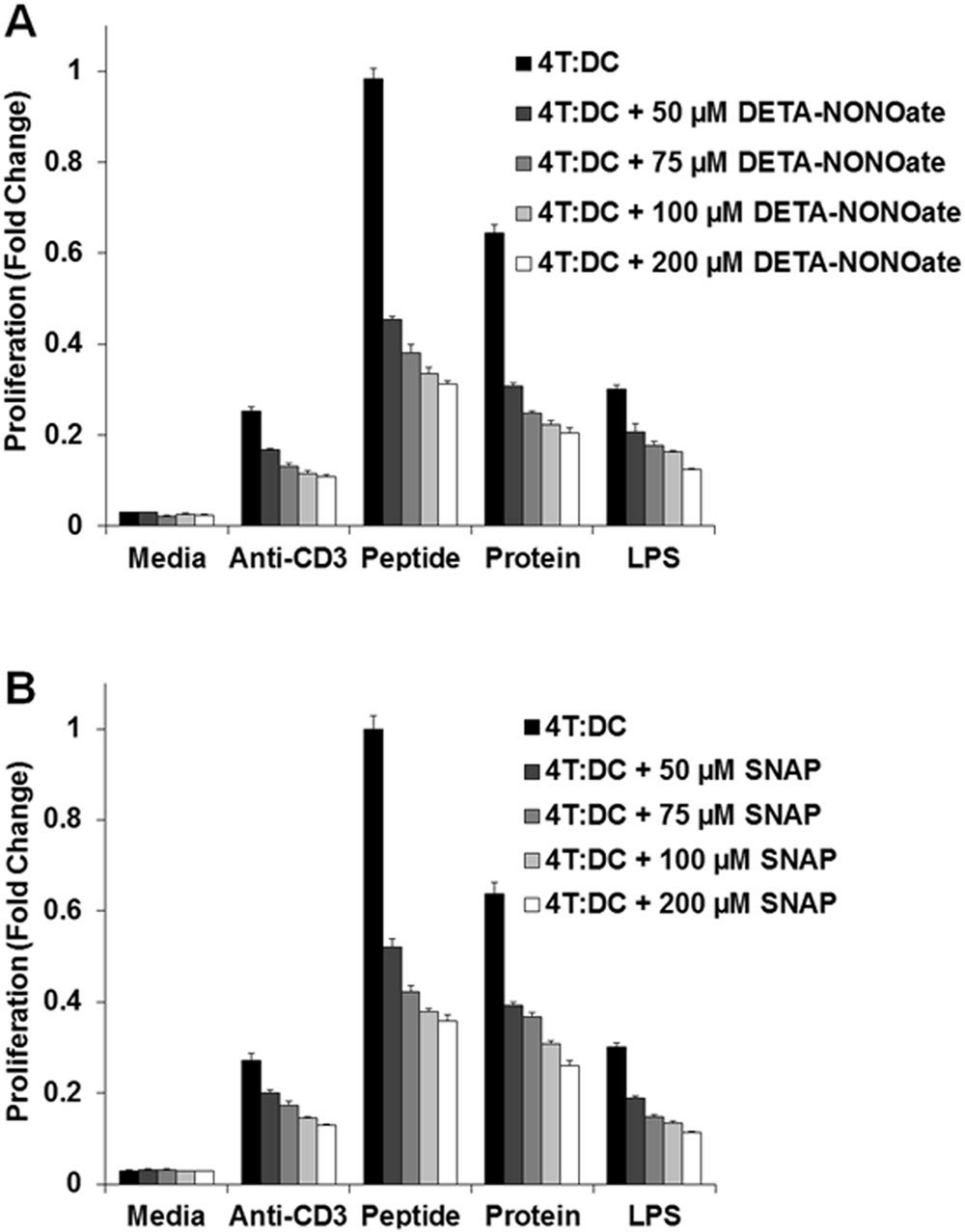
NO decreases antigen presentation from DCs to CD4^+^ T cells. The effects of NO on DC function were evaluated using transgenic mice that express a T cell receptor (TCR) specific for a defined antigen, namely ovalbumin (OVA). DCs and CD4^+^ T cells purified from the spleen and lymph nodes of an OT-II mouse were treated with anti-CD3 (global T cell activating treatment), the OVA 329–337 peptide, or whole OVA protein in the presence or absence of NO donors DETA-NONOate (panel A) or SNAP (panel B). Lipopolysaccharide (LPS) is a control for nonspecific inflammation. The data was log transformed and analyzed with one way ANOVA with the Bonferroni correction.

The effects of NO on dendritic cell (DC) function were evaluated using transgenic mice that express a T cell receptor (TCR) specific for a defined antigen, namely ovalbumin (OVA). DCs and CD4^+^ T cells purified from the spleen and lymph nodes of an OT-II mouse and cultured at a ratio of 4 T cells for every 1 DC (4 T:1DC) were treated with the OVA 329–337 peptide, anti-CD3, whole OVA protein or lipopolysaccharide (LPS) in the presence or absence of the NO donors SNAP or DETA-NONOoate. The data was log transformed and analyzed with one way ANOVA with Bonferroni correction. As seen in Fig. 1, the proliferation measured for the control 4 T:DC condition was greater than that seen following addition SNAP or DETA-NONOate at any concentration (p < 0.001). The NO donors inhibited DC antigen presentation in a dose dependent fashion. For instance when 50 µM DETA-NONOate or SNAP was compared to 100 µM DETA-NONOate or SNAP the proliferation was decreased by 0.737-fold (95% CI (0.665, 0.817), p < 0.001), and 0.727-fold (95% CI (0.659, 0.801), p < 0.001), respectively. Similar observations can be made when comparing the 200 µM and 50 µM doses. The experiments included the use of a T cell proliferation stimulus (anti-CD3) and demonstrated that NO decreased T cell activity as has been demonstrated before in the literature (Nagaraj *et al*., 2007), but the effect in the context of antigen presentation is much greater. In addition, single T and DC cell controls were performed and demonstrated low levels of T cell proliferation outside the context of antigen presentation (Supplemental Fig. 1). When the ratio of T cells to DCs was increased to 8:1 after addition of 75 µM SNAP, the level of antigen presentation decreased but there was still a trend of NO dependent inhibition of antigen presentation from DCs to CD4^+^ T cells (15.2 ± 5.7% decreased, Supplemental Fig. 2). These experiments suggest that NO has a negative effect on antigen presentation in this model.

### MDSC phenotyping by flow cytometry confirmed increased levels of NO producing MDSCs in patients with advanced disease

**Figure 2:**
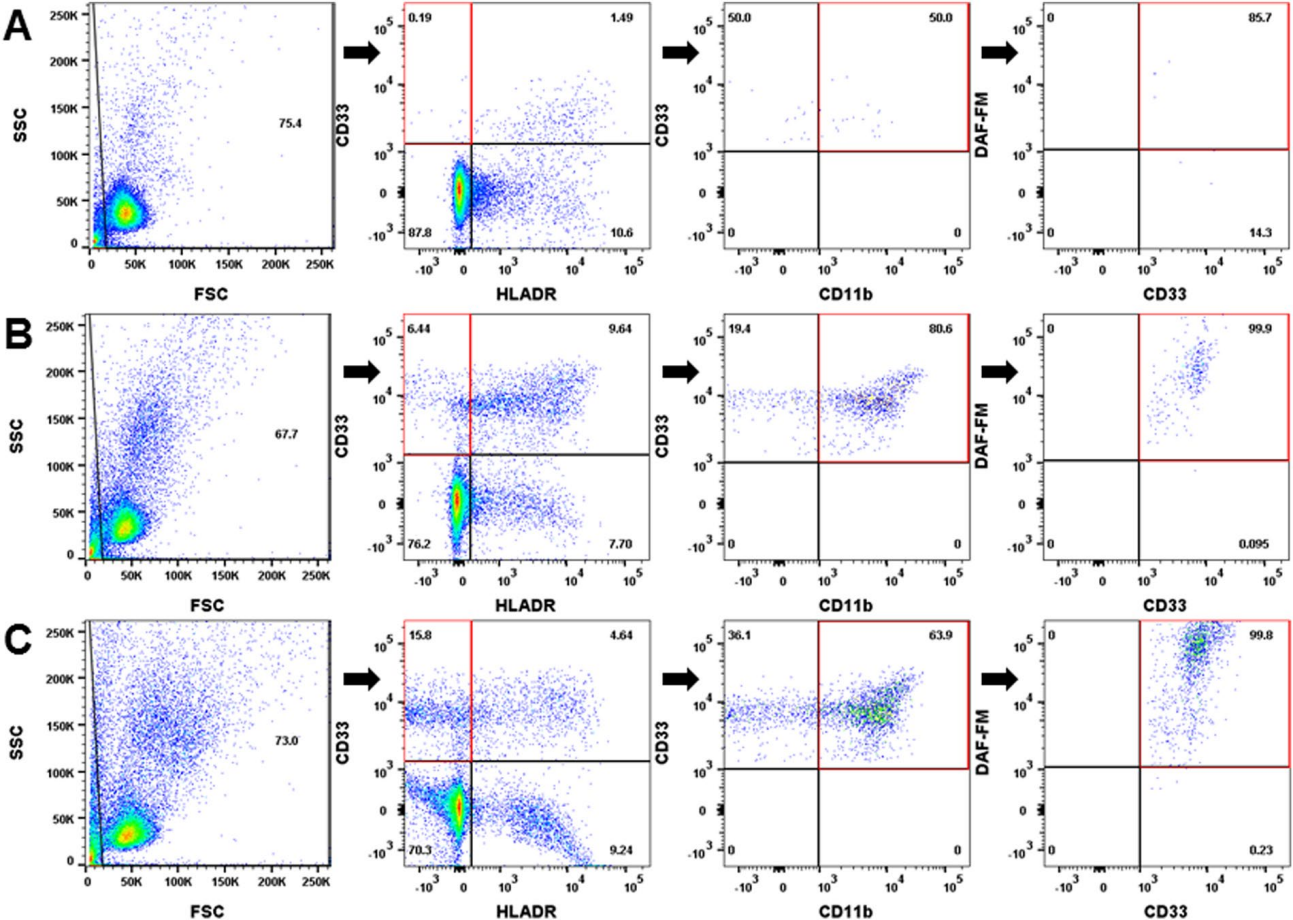
Normal PBMC have few cells with the MDSC phenotype and reduced levels of NO. (**A**) Normal PBMC derived from the peripheral blood of a normal donor were gated for HLADR^neg^CD11b^+^CD33^+^ cells. Right panel – the same cells stained for NO using DAF-FM. (**B**) PBMC derived from the peripheral blood of a melanoma patient were gated for HLADR^neg^CD11b^+^CD33^+^ cells. Right panel - The same cells stained for NO using DAF-FM demonstrating that NO is produced by human MDSCs derived from melanoma patients. (**C**) NO is also produced by HLA-DR^−^CD11b^+^CD33^+^ MDSCs derived from the blood of pancreatic cancer patients.

PBMC collected from the peripheral blood of melanoma patients and pancreatic adenocarcinoma patients were phenotyped for MDSC using the cell surface markers HLA-DR, CD33, CD11b (30,000 events) and the production of NO was measured using the DAF-FM stain. As seen in Fig. 2, MDSC (HLA-DR^neg^CD33^+^CD11b^+^) derived from melanoma and pancreatic cancer patients produce NO. Normal PBMC have few cells with the MDSC phenotype (0.19%) and those that are present produce less NO (Fig. 2, Supplemental Table 1). As human MDSC are known to release NO into the surrounding environment^14,23^, we investigated whether addition of MDSC to the co-cultures of T and DCs would decrease the levels of antigen presentation.

### MDSC decrease antigen presentation from DCs to CD4^+^ T cells via a NO dependent mechanism

**Figure 3:**
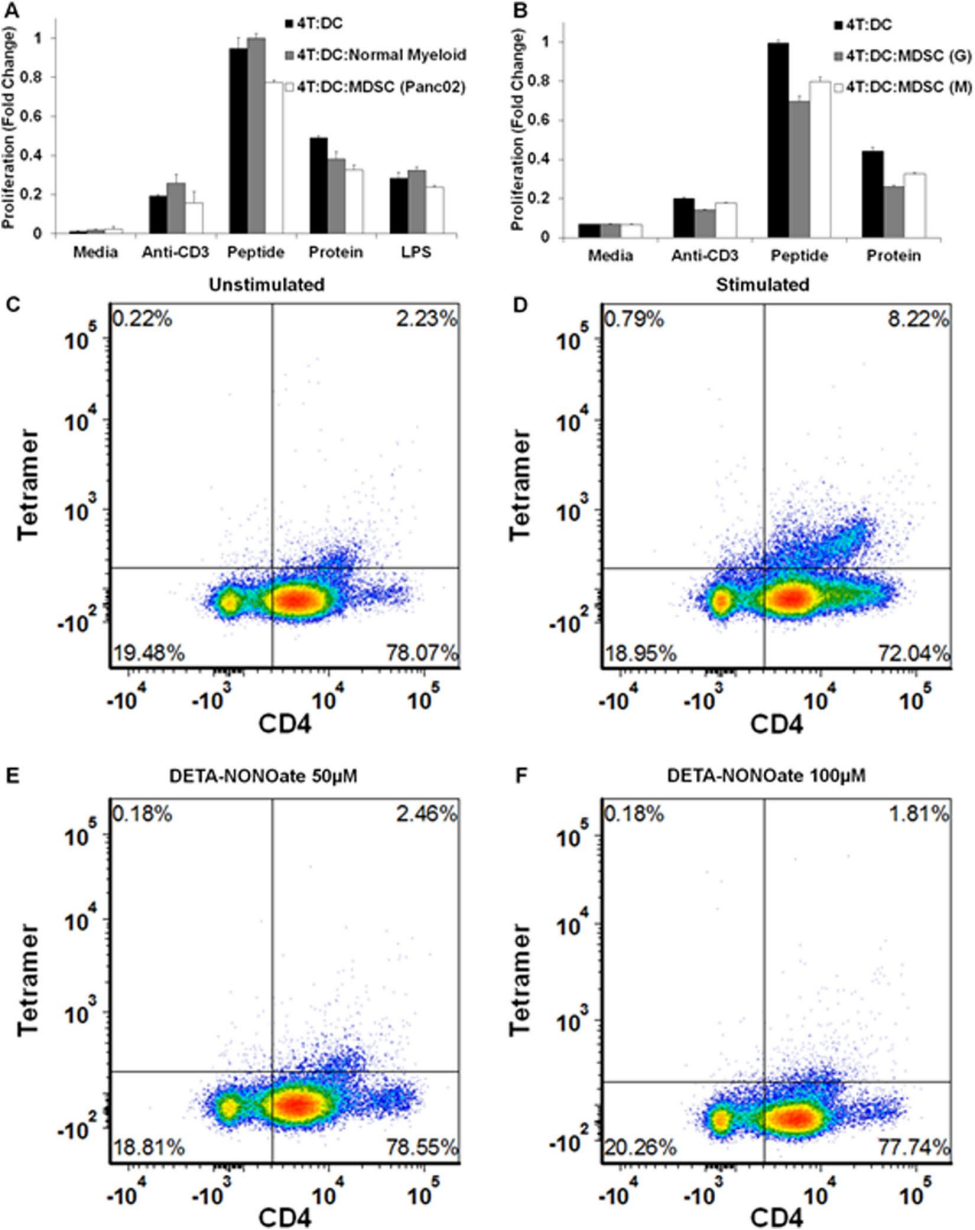
MDSC decrease antigen presentation from DCs to CD4^+^ T cells. (**A**) DCs and CD4^+^ T cells purified from the spleen and lymph nodes of an OT-II mouse were treated in the presence or absence of MDSC (granulocytic + monocytic) obtained from a pancreatic cancer model and normal myeloid cells. Myeloid cells obtained from untreated C56BL/6 mice did not suppress proliferation in the antigen presentation assay. (**B**) DCs and CD4^+^ T cells were treated in the presence of granulocytic or monocytic MDSC obtained from the murine melanoma model and demonstrated increased inhibition of antigen presentation (p < 0.001, ANOVA with Bonferroni correction). (**C**–**F**) The ability of T cells derived from the OTII mouse to detect OVA specific tetratmers was measured in the presence and absence of the nitric oxide donor, DETA-NONOate. Specifically T cells were stimulated with 10 µg/mL OTII peptide cultured for 48 hours. After 48 hours OTII specific tetramers were measured on CD3^+^CD4^+^ T cells. (**C**) Unstimulated, (**D**). Stimulated for 48 hours without DETA-NONOate, (**E**). Stimulated for 48 hours with 50 µM DETA-NONOate, and (**F**) Stimuated for 48 hours with 100 µM DETA-NONOate. Each experiment was performed in duplicate utilizing cells from different OTII mice and demonstrated similar results. A representative plot is presented for each condition. Addition of 50 µM DETA-NONOate demonstrated 68.3 ± 1.5% decrease in the ability of the TCR to recognize OTII specific antigen. Addition of 100 µM DETA-NONOate demonstrated 76.5 ± 1.9% decrease in the ability of the TCR to recognize OTII specific antigen. The viability of the cells was not significantly altered as demonstrated in (Supplemental Fig. 3).

DCs and CD4^+^ T cells purified from the spleen and lymph nodes of an OT-II T cell receptor transgenic mouse were treated in the presence or absence of MDSC or normal myeloid cells. MDSC were generated from two well-established models for studying immune reactions in cancer (pancreatic adenocarcinoma model consisting of C56BL/6 mice bearing the Panc02 tumor line, and melanoma model consisting of C56BL/6 mice bearing the B16-F10 tumor line). As seen in Fig. 3A, addition of MDSC derived from mice containing Panc02 tumors to the DC:T cell co-cultures led to a reduction in T cell proliferation (19.5 ± 6.0%, n = 3; Fig. 3A) in response to the OVA peptide presentation from DC to CD4^+^ T cells. As a control, normal myeloid cells were isolated utilizing the same phenotypic markers as MDSC isolated from C56BL/6 mice with pancreatic or melanoma cancers. These normal myeloid cells did not suppress proliferation in the antigen presentation assay (Fig. 3A). Granulocytic or monocytic MDSC derived from mice containing B16-F10 melanoma tumors appear to suppress proliferation in the setting of peptide presentation from DC to CD4+ T cells by 30.3 ± 2.9% and 20.3 ± 2.2%, respectively (Fig. 3B). The other stimuli utilized in the experiment are utilized as controls. Anti-CD3 non-specifically stimulates T cells, and the whole ovalbumin peptide needed to be processed prior to presenting the OTII peptide.

Given that NO may directly inhibit cell cycle progression and hence proliferation at sufficiently high concentrations, we tested whether NO may directly inhibit the functionality of the T-cell receptor^26^. The ability of CD4^+^ T cells derived from the OTII mouse to detect OVA specific tetramers was measured in the presence and absence of the nitric oxide donor, DETA-NONOate. Specifically mouse splenocytes were stimulated with 10 µg/mL OTII peptide during a 48 hour culture. After 48 hours OTII specific tetramers were used to analyze CD3^+^CD4^+^ T cells that were unstimulated, stimulated for 48 hours without DETA-NONOate, stimulated for 48 hours with 50 µM DETA-NONOate, and stimulated for 48 hours with 100 µM DETA-NONOate (Fig. 3C–F). Each experiment was performed in duplicate utilizing cells from different OTII mice and demonstrated similar results. A representative plot is presented for each condition. The decrease in recognition of OT II tetramers was 68.3 ± 1.1% and 76.5 ± 1.3% at 50 and 100 µM DETA-NONOate, respectively (Fig. 3C–F). The viability of the cells was not significantly altered as demonstrated in Supplemental Fig. 3.

**Figure 4:**
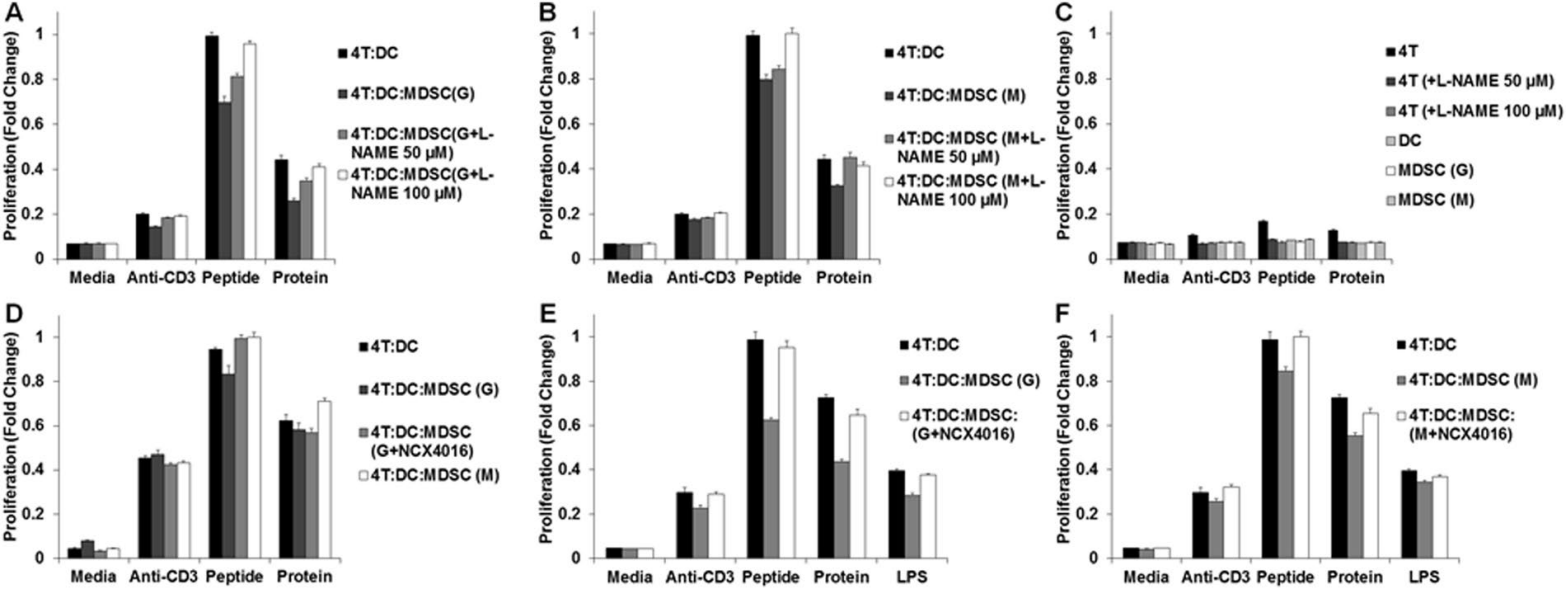
MDSC inhibition of antigen presentation from DCs to T cells is abrogated in the presence of a nitroaspirin. DCs and CD4^+^ T cells purified from the spleen and lymph nodes of an OT-II mouse were treated in the presence or absence of granulocytic or monocytic MDSC derived from the B16-F10 or Panc02 murine models. (**A**,**B**) Addition of granulocytic MDSC (**A**) or monocytic MDSC (**B**) obtained from the melanoma model (B16-F10) to the co-culture of T cells and DCs reduced proliferation by (0.70 fold of control (p < 0.001, 95% CI (0.64, 0.76), one-way ANOVA with Bonferroni correction), (0.80 fold of control (p < 0.001, 95% CI (0.74, 0.86), one-way ANOVA with Bonferroni correction), respectively. When granulocytic or monocytic MDSC were pre-treated with L-NAME and then added to the DC-T cell co-culture, proliferation in the setting of antigen presentation of DC to CD4+ T cells was restored (p < 0.001, p < 0.001), respectively. (**C**) T,DC, and MDSC single cell controls for antigen presentation demonstrate limited proliferation. (**D**) Addition of granulocytic MDSC obtained from a pancreatic cancer Panc02 murine model to the co-culture of T cells and DCs trended to reduced proliferation by 0.88 fold of control (p = 0.065; 95% CI (0.79, 0.97); one-way ANOVA with Bonferroni correction). When MDSC were pre-treated with a nitroaspirin and then added to DC-T cell co-culture, proliferation in the setting of antigen presentation of DC to CD4^+^ T cells was restored (p = 0.013; one-way ANOVA with Bonferroni correction). Addition of monocytic MDSC demonstrated a variable effect on proliferation of the DC-T co-culture. (**E**,**F**) Addition of granulocytic MDSC (**E**) or monocytic MDSC obtained from the melanoma model (B16-F10), to the co-culture of T cells and DCs reduced proliferation as seen in (**A**,**B**). When granulocytic or monocytic MDSC were pre-treated with NCX-4016 and then added to the DC-T cell co-culture, proliferation in the setting of antigen presentation of DC to CD4+ T cells was restored for both the granulocytic subset (p < 0.001), and the monocytic subset (p = 0.015), respectively.

Given the proliferation data and the tetramer binding data demonstrating NO inhibition of antigen presentation, DCs and CD4^+^ T cells purified from the spleen and lymph nodes of an OT-II mouse were treated in the presence or absence of granulocytic or monocytic MDSC and two different iNOS inhibitors. MDSC in this system were first treated with a nitric oxide inhibitor, the inhibitor was washed away, and then the MDSC were added to the 4 T:DC co-culture. Addition of granulocytic or monocytic MDSC obtained from the melanoma model (B16-F10) to the co-culture of T cells and DCs reduced proliferation in the presence of peptide by 0.70-fold compared to control (p < 0.001, 95% CI (0.64, 0.76), one-way ANOVA with Bonferroni correction) and 0.80-fold (p < 0.001, 95% CI (0.74, 0.86), one-way ANOVA with Bonferroni correction), respectively (Fig. 4A,B). When granulocytic or monocytic MDSC were pre-treated with L-NAME, a known iNOS inhibitor, and then added to the DC-T cell co-culture, proliferation in the setting of antigen presentation of DC to CD4+ T cells was restored (p < 0.001, p < 0.001), respectively (Fig. 4A,B). When T, DC, and MDSC single cell controls were exposed to peptide for antigen presentation they demonstrate limited proliferation, as expected (Fig. 4C). It was confirmed that the intraperitoneal route of administration of Panc02 cells also produced MDSC with similar suppressive activity as the subcutaneous grown tumors on antigen presentation with the OTII peptide (Fig. 4D). Addition of granulocytic MDSC obtained from a pancreatic cancer Panc02 murine model to the co-culture of T cells and DCs trended to reduced proliferation in the presence of the OT II peptide by 0.88 fold of control (p = 0.065; 95% CI (0.79, 0.97); one-way ANOVA with Bonferroni correction). A second iNOS inhibitor NCX-4016 (nitroaspirin) tends to inhibit iNOS by turning off iNOS mRNA expression^27^. When MDSC were pre-treated with a NCX-4016 and then added to DC-T cell co-culture, proliferation in the setting of antigen presentation of DC to CD4^+^ T cells was restored (p = 0.013; one-way ANOVA with Bonferroni correction). Addition of monocytic MDSC demonstrated a variable effect on proliferation of the DC-T co-culture. All figures are a representative graph of two independent experiments. The melanoma antigen presentation experiments from above were duplicated using NCX-4016 and similar results were seen for decreased proliferation in the setting of peptide stimulation (Fig. 4E,F). When granulocytic or monocytic MDSC were pre-treated with NCX-4016 and then added to the DC-T cell co-culture, proliferation in the setting of antigen presentation of DC to CD4+ T cells was restored for both the granulocytic subset (p < 0.001), and the monocytic subset (p = 0.015), respectively (Fig. 4E,F). As antigen presentation is dependent on STAT1 phosphorylation and it was previously shown that STAT1 nitration prevents STAT1 phosphorylation at amino acid position 701^3,10^, a novel mass spectrometry assay was developed to measure nitrated STAT1 in PBMC collected from patients with advanced cancer.

### Measurement of nitrated and native STAT1 protein

**Figure 5:**
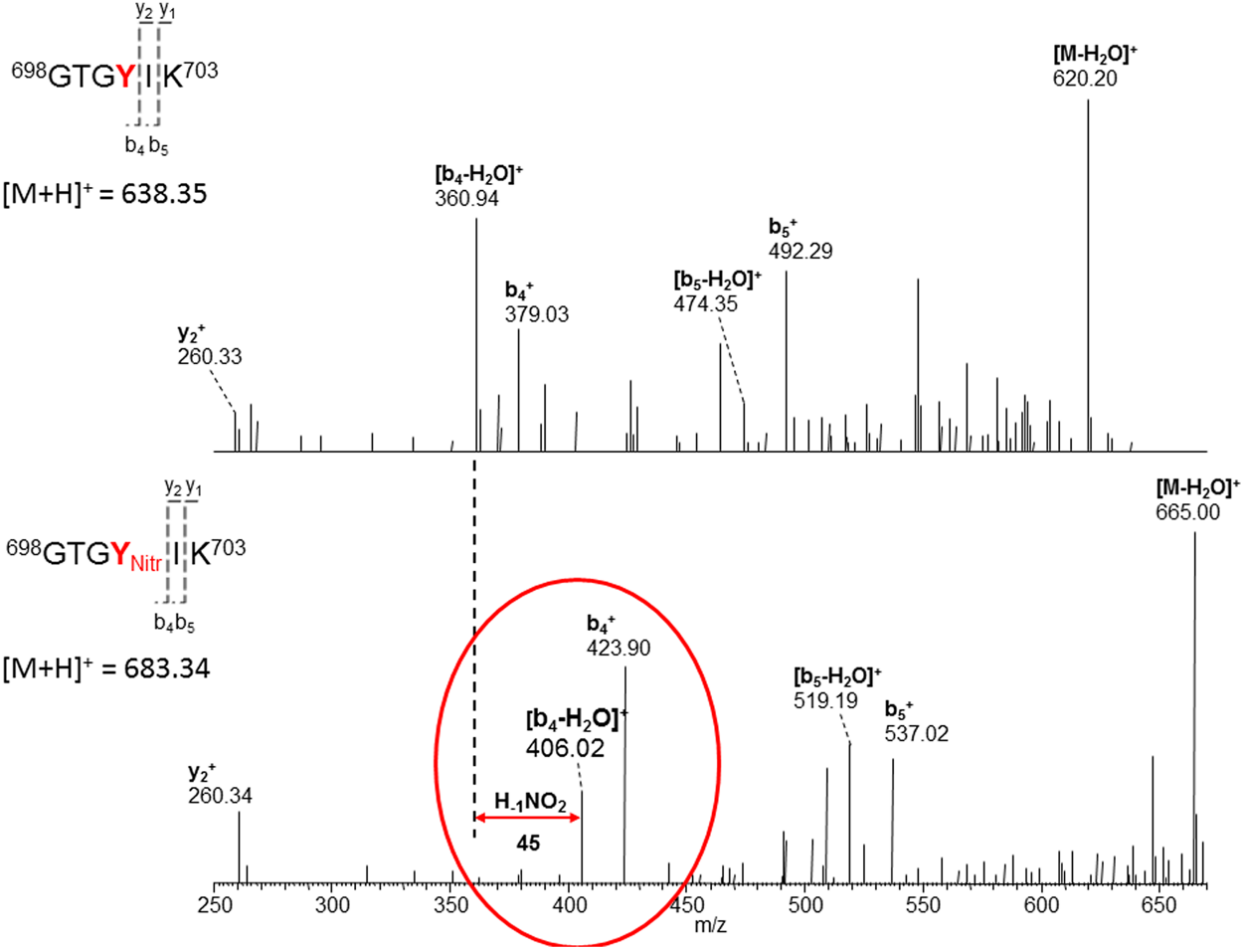
Mass spectrum of a peptide from STAT1 containing Tyr701. Top panel – Full length STAT1 protein was treated with peroxynitrite and subjected to trypsin cleavage. The resulting peptides were run on the HPLC and Orbitrap mass spectrometer. The HPLC and mass spectra of the native peptide are presented. Bottom panel - Spectrum of NO-treated STAT1 (GTGnYIK). There is a shift in the retention time and m/z ratio observed for nitrated Tyr701. The fragmentation pattern important for identifying the nitration moiety is demonstrated in the figure. Labeling for N-terminal or “b” fragments and C-terminal or “y” fragments is consistent with standardized nomenclature in proteomics research. Please refer to^55^ for more details on this nomenclature.

The full length STAT1 protein (5 µg; purity >95%) was digested with proteolytic sequencing grade enzymes consisting of either trypsin or Glu C at varying ratios of digestion enzyme to STAT1 protein (1:10–1:50). The goal was to find the optimal peptide containing the tyrosine 701 residue for directed mass spectrometry experiments. The samples were then run on a capillary-liquid chromatography-nanospray tandem mass spectrometer to identify the peptides associated with proteolytic enzyme (Trypsin, Glu C) digestion of the full length STAT1 protein. Trypsin digestion was performed on the STAT1 protein for various digestion times, and it was observed that optimal digestion occurs at greater than ~16 hours. The peptide observed on the mass spectrometer associated with the tyrosine at position 701 of STAT1 is GTGYIK (amino acid residues 698–703, Fig. 5). Given that the trypsin digestion only produced a peptide that was 6 amino acids in length, digestion with the sequencing grade enzyme Glu C was also attempted. However, a peptide containing the Y701 residue was not observed using Glu C digestion. Therefore, sequencing grade trypsin was utilized to digest all the samples presented in this report. Subsequently, full length STAT1 protein (>95% pure) was treated with peroxynitrite to nitrate tyrosine residues on the purified STAT1 protein and subjected to trypsin digestion to measure the nitrated form of tyrosine at position 701. The resulting nitrated peptides were run on the liquid chromatography/ mass spectrometer system as before. When the tyrosine at position 701 is nitrated there are increases in the retention time on the column and the and m/z ratio on the mass spectrometer (Fig. 5 and Supplemental Fig. 4).

**Figure 6:**
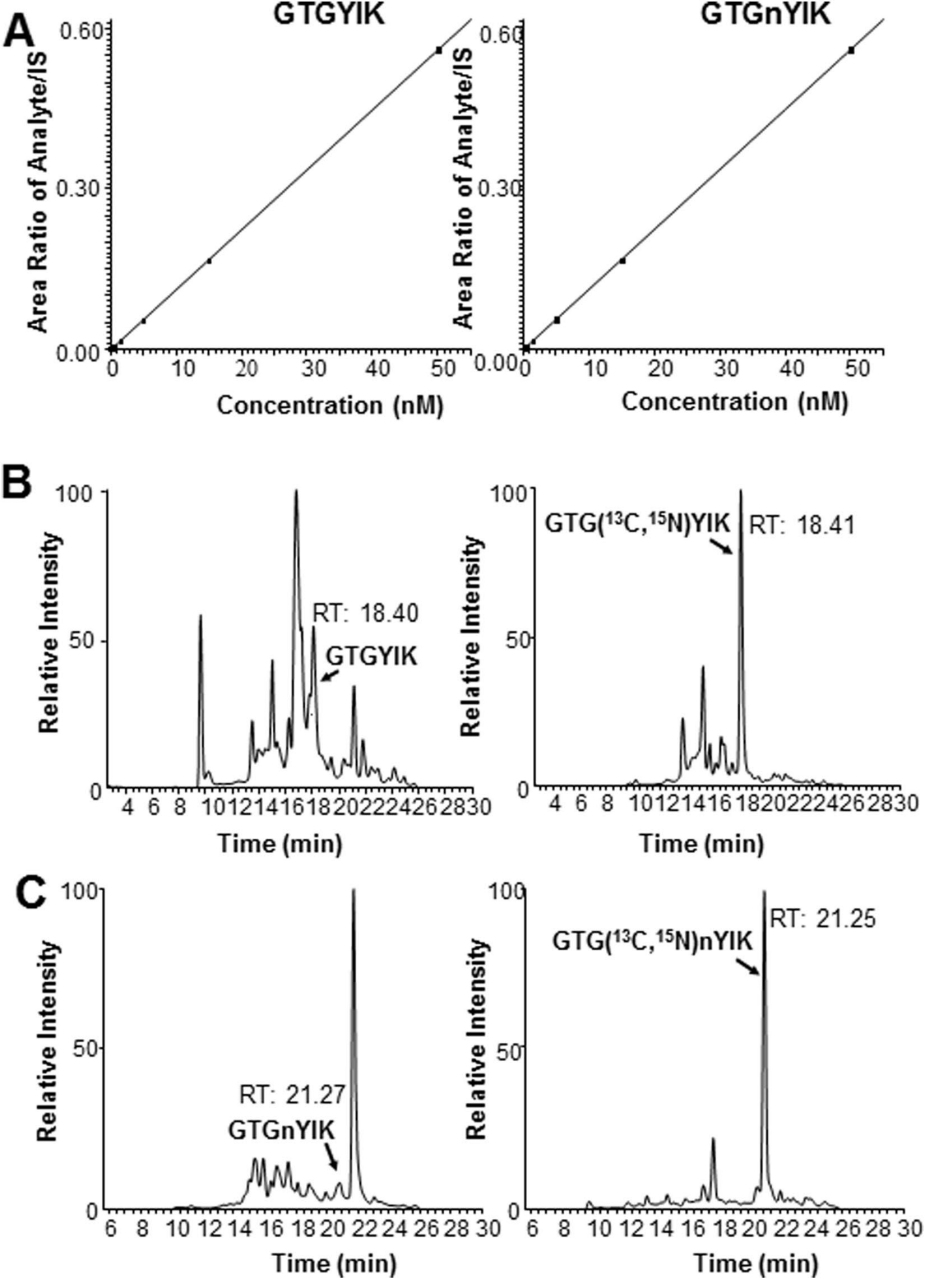
Mass Spectrometry SRM experiments detects both the native STAT1 (GTGYIK) and nitrated STAT1 (GTGnYIK) at position 701. (**A**) Calibration curves of GTGYIK from 0.05–50 nM R^2^ = 0.9996 (STAT1), R^2^ = 0.9961 (nSTAT1). (**B**) SRM chromatograms of GTGYIK, and its isotopic peptides containing ^13^C and ^15^N in the amino acid glycine at position 3 of the peptide in PBMC control samples. (**C**) SRM chromatograms of GTGnYIK and isotopic peptide spike-in GTG(^13^C, ^15^N)nYIK in PBMC control samples.

The LC-MS/MS assay developed to quantify the GTGYIK peptide from STAT1 was employed to measure STAT1 and nitrated STAT1 levels in patient PBMC samples. Mass spectrometry SRM experiments detected both the native STAT1 (GTGYIK) and nitrated STAT1 (GTGnYIK) at position 701. Calibration curves of GTGYIK, GTGnYIK, GTG(^13^C, ^15^N)YIK, and GTG(^13^C, ^15^N)nYIK were constructed from 0.05–50 nM (Fig. 6; R^2^ > 0.97). The linear range of both peptides was 0.05 to 50 nM (Fig. 6). At this point, the synthetic isotopic peptide GTG(^13^C, ^15^N)YIK, or GTG(^13^C, ^15^N)nYIK was spiked into a sample of peptides derived from PBMC obtained from a normal donor. Using the signal intensity of the isotopic peptides and the calibration curves, the concentration of STAT1 was determined. As a control, STAT1 from 3 independent PBMC samples treated with SNAP (NO donor) measured via SRM experiments demonstrated an approximately 2.06 ± 0.39 fold increase in the level of nitrated STAT1 at amino acid position 701 (Supplemental Table 3).

**Figure 7:**
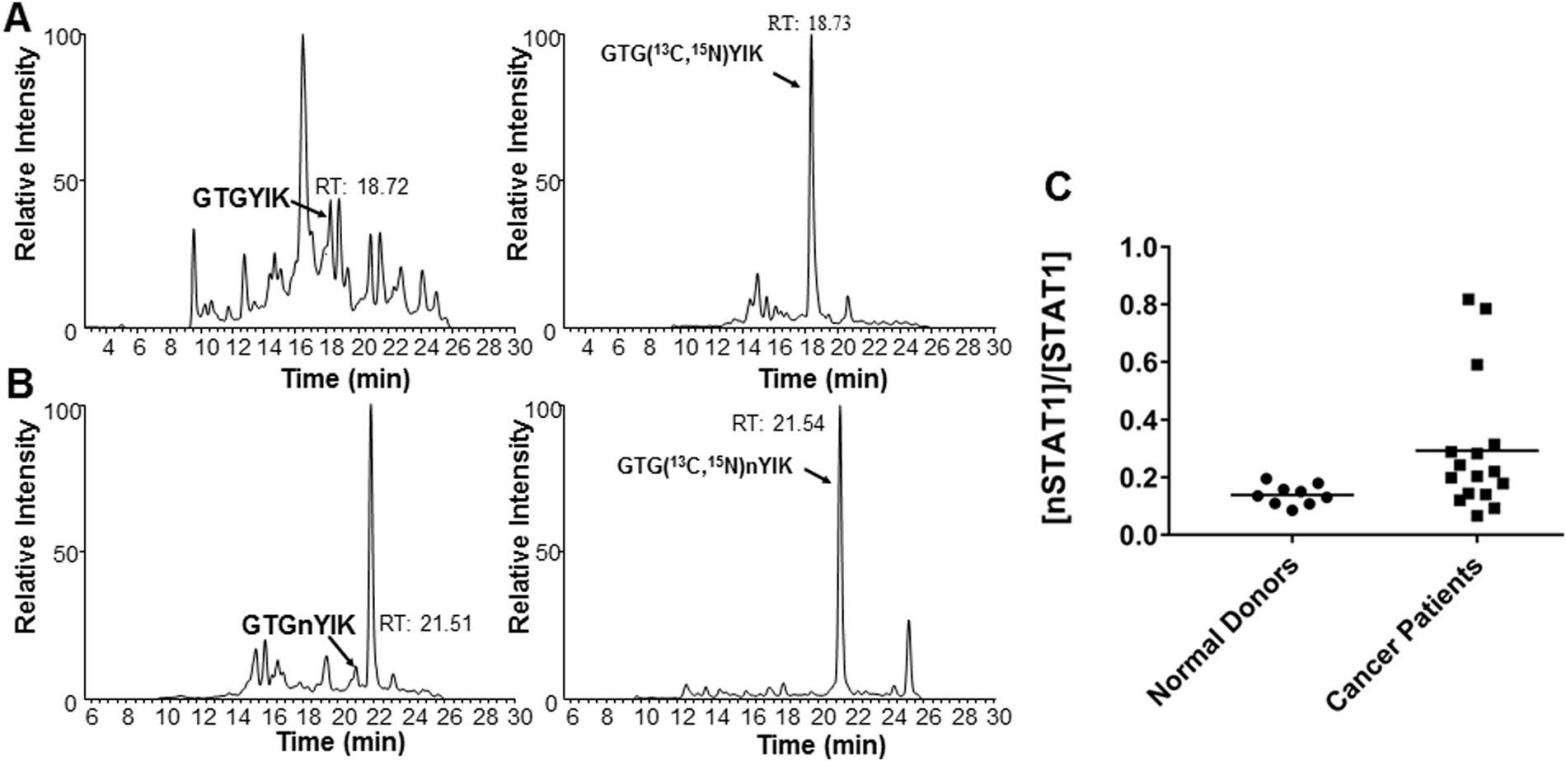
STAT1 is nitrated in melanoma and pancreatic cancer patients. (**A**) SRM chromatograms of GTGYIK and their isotopic peptide spike-in GTG(^13^C, ^15^N)YIK in PBMC obtained from patients. (**B**) SRM chromatograms of GTGnYIK (nitrated) and their isotopic peptide spike-in GTG(^13^C, ^15^N)nYIK in PBMC obtained from patients. (**C**) Measurement of the ratio of the concentration of GTGnYIK to the concentration of GTGYIK for 9 normal donors (0.14 ± 0.036) and 16 cancer patients (8 melanoma patients and 8 pancreatic cancer patients, 0.30 ± 0.24), p = 0.036, T test).

Mass spectrometry SRM experiments detected both the native STAT1 (GTGYIK) and nitrated STAT1 (GTGnYIK) peptides at position 701. Afterwards, PBMC derived from the peripheral blood of 8 melanoma patients, 8 pancreatic cancer patients, and 9 normal donors were prepared for a nitrated STAT1 quantification experiment. All of the cancer patients had metastatic disease, but varied in regards to type of therapy being administered. As seen in Fig. 7, STAT1 is nitrated in the PBMC of melanoma and pancreatic cancer patients. SRM chromatograms of GTGYIK and GTGnYIK measured in PBMC obtained from a melanoma patient are shown in Fig. 6 alongside the isotopic spike-in control peptides (Fig. 7A,B). To quantify the relationship between the nitrated STAT1 peptide and the native STAT1 peptide the measurement of the ratio of the concentration of [GTGnYIK] to the concentration of [GTGYIK] was plotted for the 9 normal donors, 8 melanoma patients and 8 pancreatic cancer patients (Fig. 7C, Supplemental Fig. 5). The ratio of [nSTAT1]/[STAT1] was increased in cancer patients (0.30 ± 0.24; melanoma and pancreatic cancer) as compared to normal controls (0.14 ± 0.04, p = 0.036, T test). Anecdotally, the patients with the highest disease burden also had the highest [GTGnYIK]/[GTGYIK] ratio (Ratio > 0.4; Fig. 7C). For instance, the pancreatic cancer patient who had the highest [GTGnYIK]/[GTGYIK] ratio expired within two months of peripheral blood collection. This data suggests that there is a trend towards higher nSTAT1 levels in cancer patients. The use of specific cut-off values for biomarker purposes will be the subject of future investigations.

## Discussion

In this study, we have demonstrated that antigen presentation from dendritic cells to CD4^+^ T cells is inhibited by NO. Interestingly, multiple groups have demonstrated that a class of inhibitory immune cells called MDSC produce NO^1,15,16^. As such, we demonstrated that MDSC also inhibit antigen presentation from dendritic cells to CD4^+^ T cells and that this inhibition was abrogated by treatment of MDSC with iNOS inhibitors. It has been shown that STAT1 protein is important for antigen presentation and that STAT1 is nitrated by MDSC^3,4,10^. Therefore, we developed a method to measure the nitration of STAT1 within PBMC collected from cancer patients.

Myeloid cells were first found to have suppressive effects on T cells that led to blunting of immune responses. Both the wild type and inducible NO synthase (iNOS) deficient mice had comparably increased numbers of CD11b^+^Gr-1^+^ cells (now known as MDSC) after treatment with granulocyte macrophage colony-stimulating factor (GM-CSF). However, immunosuppression, as measured by inhibition of T cell proliferation, was observed only in wild type mice thus establishing a role for NO mediated inhibition of immune responses^28^. MDSC have immune suppressive effects on host immune cells and thereby help cancers evade the immune response^29–31^. Initially, in lymphoma models, iNOS was found to be highly expressed in monocytic MDSC^32^. It is generally thought that NO produced by iNOS may produce reactive nitrogen species (e.g. peroxynitrite) and result in apoptosis of T cells^33^. Other cellular targets are also potentially subject to nitration such as the T cell receptor^34–36^. In the CT26 colon cancer model the presence of nitration of the CCL2 chemokine diminished CD8^+^ T cell infiltration and may attract MDSC into the tumor microenvironment^37^. Treatment with the Bruton Tyrosine Kinase inhibitor, ibrutinib, in human PBMC and in a B16 murine melanoma model led to decreased NO production and inhibition of MDSC generation^38^. In these studies, NO derived from MDSC inhibited antigen presentation from DC to T cells.

It is widely accepted that type I interferon signaling through the JAK/STAT signaling pathway is an important mechanism by which the host immune system recognizes tumors^39^. STAT1 has been demonstrated to be a key mediator in antigen presentation and the host defense against multiple tumor types^3,4^. Our group has identified that this signaling pathway is disrupted by decreased phosphorylation and increased NO mediated nitration of the STAT1 protein^15^. Tyr-701 phosphorylation stabilizes SH2 domain dimerization and increases STAT1 DNA binding by more than 200-fold promoting STAT1 signal transduction^40^. STAT1 dimers are known to translocate to the nucleus and activate interferon response pathways. In murine models lacking a functional STAT1 gene, immune cells cannot respond to exogenous interferon α administration^8^. In the current study, we demonstrate that nitration can occur at position 701 of the STAT1 protein in PBMC derived from both pancreatic cancer and melanoma patients.

Selective reaction monitoring (SRM) or multiple reaction monitoring (MRM) mass spectrometry was utilized to measure nitration of the STAT1 peptide. A peptide fragment needs to be identified to develop the selective reaction monitoring experiment (synonym multiple reaction monitoring) to detect that specific peptide from patient samples. Selecting the SRMs to monitor is a challenge for any given protein. SRM/MRM methods without isotopic labeling of the atoms in the peptide can be used for protein identification and quantification (similar to an ELISA). However, multiple peptides per protein were needed to perform the assay^41^. In addition, the detection of the transitions of one peptide ion fragment to a smaller peptide fragment in the mass spectrometer can be developed experimentally or with prediction programs such as MRMaid^42^. Large scale SRM efforts have been undertaken. In one such effort 319 proteins important in breast cancer were studied and the MRM data corresponds to the molecular subtyping of luminal and basal subtypes^43^. Clinically, the SRM can measure proteins quantitatively, but the method is limited to high and moderately abundant proteins (31 mg/mL to 44 ng/mL)^20,44^. Methods have also been developed recently with isotopically labeled peptides to measure other post-translational modifications such as histone propionylation ranging from µM to nM scales^45^. In the current case, automated detection of a random peptide within STAT1 with maximum signal intensity in the SRM experiment was not possible due to the specific requirement to measure nitration at Tyr-701. In addition, the concentration of STAT1 in peripheral blood is below the limit of detection of most triple quadrupole mass spectrometers. Not only did the assay require isotopic labeling, but also required utilization of new mass spectrometry technology using the TSQ Quantiva mass spectrometer. Therefore, the current method has the potential to be applicable to cases where the amino acid for the post-translational modification is known, the abundance of the protein is in the picomolar range, and that specific modification needs to be monitored in a clinical setting.

In this study, MDSC-derived NO inhibited antigen presentation from DC to T cells and this effect can be abrogated by treating MDSCs with iNOS inhibitors. Given the potential importance of nitration of the STAT1 protein, a method for detecting peptides from PBMC samples with important post-translational modifications was developed. This method can detect nitrated STAT1 down to picoMolar concentrations in PBMC samples derived from cancer patients. Importantly, the techniques developed herein may be applied to other post-translational modifications of clinical significance.

## Methods

### Preparation of nitrated STAT1 protein for mass spectrometry

10 µg of STAT1 (purchased from Life Technologies; cat. no. PHF0011) was placed in MOPS buffer ((3-(*N*-morpholino)propanesulfonic acid)) at pH 8.0 and was nitrated using peroxynitrite (Cayman Chemical, Ann Arbor, MI) at 150 molar excess. 5 mM of 1,4-dithiothreitol was added to the solution to prevent additional oxidation. The buffer was exchanged via dialysis against 50 mM ammonium bicarbonate pH 8.0 at 4 °C. 500 ng of sequencing grade trypsin (Promega, Madison, WI; cat. no. V5111) was added to the mixture in addition to 0.1% Rapigest™ SF surfactant (Waters Milford, MA) in 50 mM ammonium bicarconate. The Rapigest™ detergent was allowed to digest the pure STAT1 protein for various periods of time from 4 hours to 24 hours. After the digestion step was complete, 30% formic acid was added to the mixture to deactivate the Rapigest™ SF surfactant by placing it at 37 **°**C for 1 hour followed by incubation at 4 °C for 1 hour. Afterwards, the samples were spun in a microcentrifuge at 14,000 rpm at 4 °C for 15 minutes. The deactivated Rapigest™ SF surfactant forms an upper layer, allowing the collection of the peptide containing fluid from below taking care not to collect any pellet that had formed at the bottom of the eppendorf tube.

### Sources of peripheral blood mononuclear cells (PBMC)

There were four sources of PBMC. Leukopaks purchased from the American Red Cross or (OneBlood, St. Petersburg, FL) were processed for PBMC via ficoll centrifugation as previously described^46^. Peripheral blood was collected from melanoma and pancreatic cancer patients under The Ohio State University Institutional Review Board (IRB) approved tumor bank protocols for the respective malignancies (OSU-0458 and OSU-06002). Informed consent was obtained from all patients. All methods were carried out in accordance with relevant guidelines and regulations. Peripheral blood from normal donors was collected under OSU-0458 and processed for PBMC via ficoll extraction as previously performed^46^.

### Antigen presentation

OTII mice were bred in the laboratory using an IACUC approved protocol at The Ohio State University or the Moffitt Cancer Center. All experiments were performed in accordance with relevant guidelines and regulations. The spleens and inguinal lymph nodes were harvested and purified for T cells and dendritic cells by magnetic bead technique per manufacturer’s instructions (Miltenyi Biotek, San Diego, CA). Cells were plated at a ratio of four T cells for every one dendritic cell (4 T:DC) and subjected to various stimuli: 3 µg/mL anti-CD3 (BD, San Jose CA), 10 µg/mL OTII peptide (Anaspec, Fremont, CA,), and 1 mg/mL whole chicken ovalbumin peptide (Sigma-Aldrich, St. Louis, MO) or 5 µg/mL LPS (Sigma-Aldrich, St. Louis, MO). The murine tumor models utilized in these experiments to obtain MDSC were pancreatic cancer (Panc02) and melanoma (B16-F10)^38,47,48^. After 48 hours, the cells were pulsed with ^3^H thymidine, harvested 18 hours later, and measured for uptake of ^3^H thymidine as previously described^49^. These experiments were repeated in the presence of a NO donor SNAP (S-Nitroso-N-Acetyl-D,L-Penicillamine; Fisher Scientific, Hampton, NH) or DETA-NONOate (Diethylenetriamine NONOate; Cayman Chemical, Ann Arbor, MI). In addition, C57BL/6 mice were injected subcutaneously with 1 × 10^6^ cells Panc02 or 1 * 10^5^ B16-F10 cells and tumors were allowed to grow for 2–3 weeks. C57BL/6 mice were also injected via the intraperitoneal route of administration with 5 × 10^5^ cells Panc02 cells and tumors were allowed to grow for 2–3 weeks. MDSC were isolated from the spleen and draining lymph nodes of these mice and added to the T and DC co-culture at a ratio of 4 T to 1 DC to 1 MDSC. MDSC were pre-treated with a nitroaspirin (NCX-4016) or L-NAME (NG-Nitro-L-arginine methyl ester hydrochloride; Tocris, Bristol, UK) and these MDSC were utilized in the T and DC co-cultures^27^. The ability of T cells derived from the OTII mouse to detect OVA specific tetramers was measured in the presence and absence of the NO donor, DETA-NONOate. Specifically T cells were stimulated with 10 µg/mL OTII peptide (Anaspec, Fremont, CA) and cultured for 48 hours. After 48 hours, OTII specific tetramers were measured on CD3^+^ CD4^+^ T cells^50,51^. Antibodies utilized included (CD3-BUV395 (BD Biosciences, San Jose, CA), CD4-Alexa Fluor 700 (BD Biosciences), T-Select I-Ab OVA 323–339 Tetramer-PE, MBL, Woburn, MA) on a LSR-II cytometer at the Moffitt Cancer Center. A live/dead stain (Zombie NIR, Biolegend, San Diego, CA) was utilized as a control to demonstrate that the NO donor was not cytotoxic to T cells.

Flow Cytometry utilizing PBMC. PBMC were stained for NO using the stain DAF-FM (Fisher Scientific, Hampton, NH) as previously described^14^. MDSCs within the PBMC were phenotypically characterized using CD33-APC, HLADR-PeCy7, CD11b-APC-Cy7 (Beckman Coulter, Brea, CA).

### Preparation of human PBMC samples for mass spectrometry analysis

Two methods were used to prepare STAT1 samples for subsequent mass spec analysis. In the first method, STAT1 was immunoprecipitated from PBMC samples using methods previously described with the modification that the digestion buffer consisted of 1% rapigest in 50 mM ammonium bicarbonate buffer^10^. After isolating STAT1 via standard gel electrophoresis and estimating the protein concentration, mass spectrometry samples were digested with trypsin and prepared for mass spectrometry as was performed for the pure STAT1 protein. The second method used to prepare PBMC was to first lyse the PBMC in 1% rapigest in 50 mM ammonium bicarbonate and then trypsinize PBMC samples for 20 hours at 37 °C. One million PBMC were assumed to contribute 25 total µg of cellular protein to the reaction as previously described^52^. Trypsin was added at a ratio of 1 µg per 20 µg of total cellular protein.

### Mass spectrometry

Two different mass spectrometry methodologies were utilized. In the first set of experiments, tandem mass spectra for peptides from the STAT1 protein that were nitrated with peroxynitrite were characterized. To accomplish this task, the prepared STAT1 peptides obtained after trypsin digestion were subjected to capillary-liquid chromatography-nanospray tandem mass spectrometry for global protein identification on a Thermo Scientific LTQ Orbitrap XL mass spectrometer equipped with a microspray source (Michrom Bioresources Inc, Auburn, CA) operated in positive ion mode. Samples were desalted and pre-concentrated on a trap column (C18, 300 μm × 5 mm, 5 µm, 100 Å, Dionex, Sunnyvale, CA) with 50 mM acetic acid for 10 minutes and then eluted onto an analytical column (0.2 × 300 mm Magic C18AQ 3 µ 200 Å, Michrom Bioresources Inc, Auburn, CA) using a Dionex UltiMate™ 3000 HPLC system. The separation was performed with a linear gradient at a flow rate of 2 µL/min using mobile phase A (0.1% formic acid in water) and mobile phase B (0.1% formic acid in ACN). Mobile phase B was increased from 2% to 50% in 130 min, then from 50% to 90% in 5 min, then kept at 90% for another 5 min before being brought back to 2% in 1 min. The column was equilibrated at 2% of mobile phase B (or 98% A) for 30 min before the next sample injection. MS data was acquired with a spray voltage of 2 kV and a standard capillary temperature of 175 °C. The scan sequence of the mass spectrometer was done using preview mode with the data dependent TopFive method: the analysis was programmed for a full MS1 scan recorded between 300–2,000 m/z and five consecutive MS/MS scans were utilized to generate product ion spectra to determine amino acid sequence of the five most abundant peaks in the MS1 spectrum.

### Peptide sequence analysis of STAT1

The raw data files collected on the mass spectrometer were converted to mzXML and MGF files by use of MassMatrix data conversion tools (version 1.3, http://www.massmatrix.net/download). Isotope distributions for the precursor ions of the MS/MS spectra were deconvoluted to obtain the charge states and monoisotopic *m/z* values of the precursor ions during the data conversion. Resulting.mgf files were searched using Mascot Daemon (version 2.3.2, Matrix Science, Boston, MA) against NCBInr human database 20111018. Trypsin or GluC was used as the enzyme and two missed cleavages were permitted. Missed cleavages are those peptides that are generated by trypsin cutting the STAT1 protein in a non-traditional location. Modifications such as oxidation (Methionine), nitration (Tyrosine) and carbamidomethylation (Cysteine) were also included in the search. The mass accuracy was set to 20 ppm for precursor ions and 0.5 Da for product ions. ^13^C peaks were also included into the search. The significance threshold was set at p < 0.05 for valid peptide identification. STAT1 peptides with a Mascot mowse score of 40 or higher containing a minimum of one unique peptide with the ion sequence tag (a, *b* or *y* ion) of five residues or better were accepted. Utilizing this algorithm, STAT1 peptides were identified and the positions of nitrated tyrosine were located.

### Development of selected reaction monitoring (MRM) assay for quantification of nitrated and non-nitrated Y701 peptides of STAT1

In the second set of mass spectrometry experiments, we specifically looked for the STAT1 peptide corresponding to the tyrosine at position 701. Selected or multiple reaction monitoring (SRM or MRM) is a technique in mass spectrometry that is utilized to measure peptides in complex mixtures with high accuracy. SRM methods have been developed to identify and quantify putative biomarkers from biological mixtures^53^. In this method, the fragmentation pattern produced by digestion of a protein with a particular protease is known and the peptides are quantified by selecting the precursor and fragmentation ions from the peptide of interest with known mass/charge (m/z) ratios^54,55^. The assay was first developed on a Dionex Ultimate 3000 RSLCnano system (capillary/micro-flow) coupled to a TSQ Quantum Ultra mass spectrometer with a CaptiveSpray ionization source. The sample was pre-concentrated (flow rate at 20 µL/min) on a PepMap100 C18 trap column and the peptide separation was carried out on an Acclaim PepMap100 C18 column with a 60 min linear gradient elution (flow rate at 2 µL/min). The MS monitoring was conducted in positive ion mode. The doubly charged ions ([M + 2 H]^2+^) of both the nitrated and native GTGYIK peptide were present as the major peaks in Q1 full scan and thus were selected as the precursor ions with transitions of m/z 319.70 → 310.65, 480.25 for [GTGYIK] and m/z 342.21.00 → 333.13, 525.24 for [GTGnYIK]. The assay lower limit of quantification (LLOQ) was 50 nM for both peptides in neat solution.

An improved method was developed on a TSQ Quantiva mass spectrometer with an Easy-spray ionization source connected to a Dionex Ultimate 3000 RSLCnano system (nano/micro-flow). The sample was pre-concentrated (flow rate of 2 µL/min) on a PepMap100 nano trap column and the peptide separation was carried out on an Easy-spray nano C18 column with a 60 min linear gradient elution (500 nL/min). The new assay had a LLOQ of 100 pM in neat solution.

### Statistical analysis

Statistical analysis was carried out by the biostatistical core at The Ohio State University. Data were log-transformed. ANOVA methods were used for modeling for the antigen presentation experiments. Fold change and the corresponding confidence intervals were presented. Significance was adjusted by the Bonferroni correction method. The mass spectrometry data were log-transformed and a t-test was utilized to compare normal donors and cancer patients.

## Electronic supplementary material

Supplemental Information
