# Author Correction: Nitric oxide mediated inhibition of antigen presentation from DCs to CD4^+^ T cells in cancer and measurement of STAT1 nitration

**DOI:** 10.1038/s41598-018-21306-z

**Published:** 2018-03-06

**Authors:** Joseph Markowitz, Jiang Wang, Zach Vangundy, Jia You, Vedat Yildiz, Lianbo Yu, Isaac P. Foote, Owen E. Branson, Andrew R. Stiff, Taylor R. Brooks, Brandon Biesiadecki, Thomas Olencki, Susheela Tridandapani, Michael A. Freitas, Tracey Papenfuss, Mitch A. Phelps, William E. Carson

**Affiliations:** 1 Moffitt Cancer Center Department of Cutaneous Oncology, Tampa, United States; 2 Department of Oncologic Sciences USF Morsani School of Medicine, Tampa, United States; 3 Comprehensive Cancer Center, The Ohio State University Wexner Medical Center, Columbus, United States; 4 Department of Biomedical Informatics, The Ohio State University Wexner Medical Center, Columbus, United States; 5 Department of Physiology and Cell Biology, The Ohio State University, Columbus, United States; 6 Division of Medical Oncology, The Ohio State University Wexner Medical Center, Columbus, United States; 7 Department of Surgery, The Ohio State University Wexner Medical Center, Columbus, United States

Correction to: *Scientific Reports* 10.1038/s41598-017-14970-0, published online 13 November 2017

The original version of this Article contained a typographical error in the spelling of the author William E. Carson, which was incorrectly given as William E. Carson 3rd. This has now been corrected in the PDF and HTML versions of the Article, and in the accompanying supplementary material.

