# Supplementary material for: Nitric oxide mediated inhibition of antigen presentation from DCs to CD4+ T cells in cancer and measurement of STAT1 nitration
Source: Sci Rep. 2017 Nov 13;7:15424. doi: 10.1038/s41598-017-14970-0 (PMC5684213; doi:10.1038/s41598-017-14970-0)
Supplement: Supplementary file 1 — Supplemental Information [file 41598_2017_14970_MOESM1_ESM.pdf]

Nitric oxide mediated inhibition of antigen presentation from DCs to $CD4^+$ T cells in cancer and measurement of STAT1 nitration

Joseph Markowitz[1,2,3], Jiang Wang[3], Zach Vangundy[3], Jia You[3], Vedat Yildiz[3,4], Lianbo Yu[3,4], Isaac P. Foote[1], Owen E Branson[3], Andrew R Stiff[3], Taylor R. Brooks[3], Brandon Biesiadecki[5], Thomas Olencki[6], Susheela Tridandapani[3], Michael A. Freitas[3], Tracey Papenfuss[3], Mitch A. Phelps[3], William E. Carson[3,7]

[1]Moffitt Cancer Center Department of Cutaneous Oncology, [2]Department of Oncologic Sciences USF Morsani School of Medicine, [3]Comprehensive Cancer Center, [4]Department of Biomedical Informatics, [5]Department of Physiology and Cell Biology, [6]Division of Medical Oncology, [7]Department of Surgery, The Ohio State University Wexner Medical Center

Supplemental Figure 1

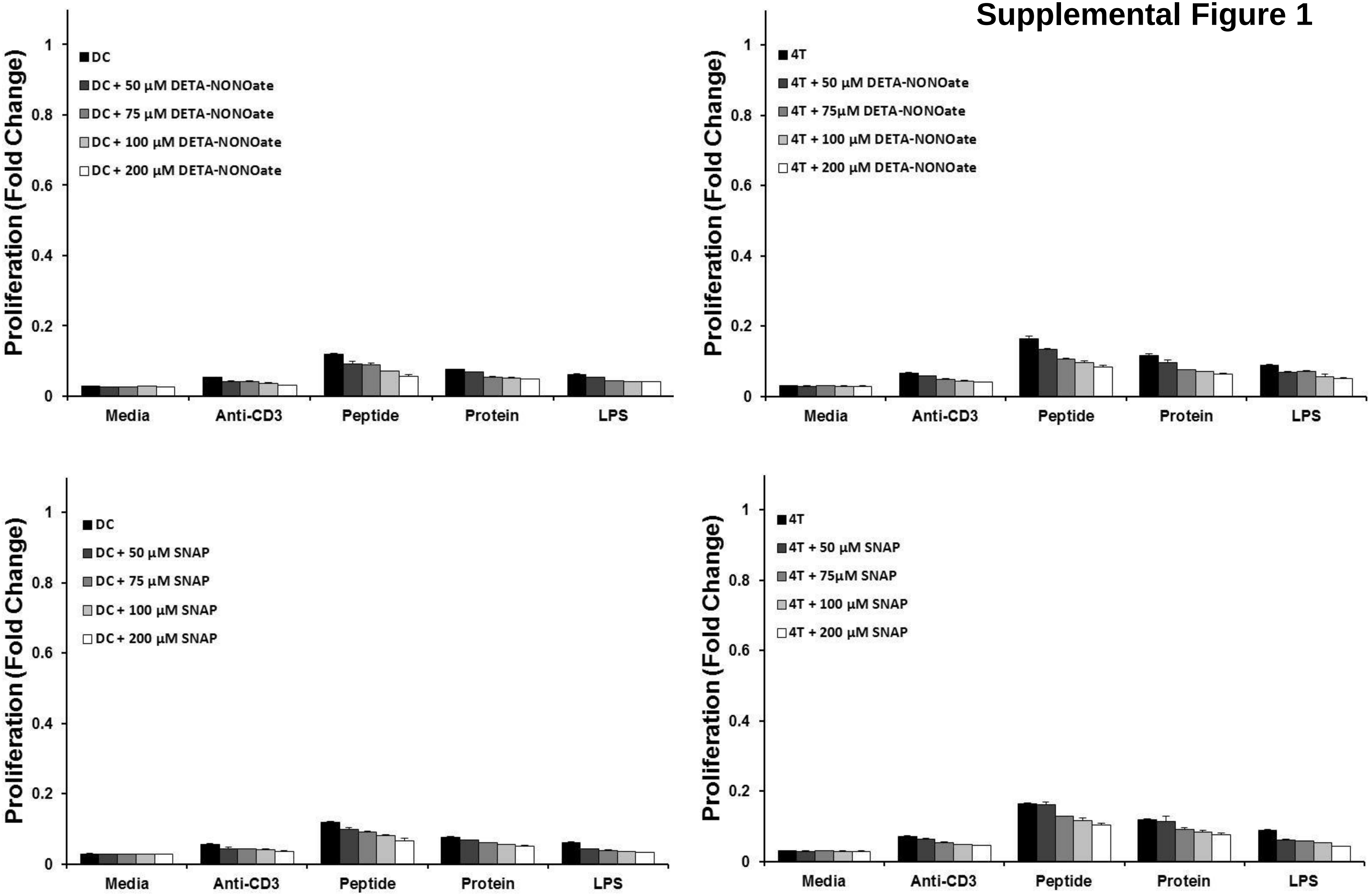

Supplemental Figure 1: The effects of NO on DC or T cell proliferation were evaluated using transgenic mice that express a T cell receptor (TCR) specific for a defined antigen, namely ovalbumin (OVA). DCs and $CD4^+$ T cells purified from the spleen and lymph nodes of an OT-II mouse were treated with anti-CD3 (global T cell activating treatment), the OVA 329-337 peptide, or whole OVA protein in the presence or absence of NO donors DETA-NONOate or SNAP. Lipopolysaccharide (LPS) is a control for nonspecific inflammation. In the absence of antigen presentation, there are low levels of proliferation.

Supplemental Figure 2

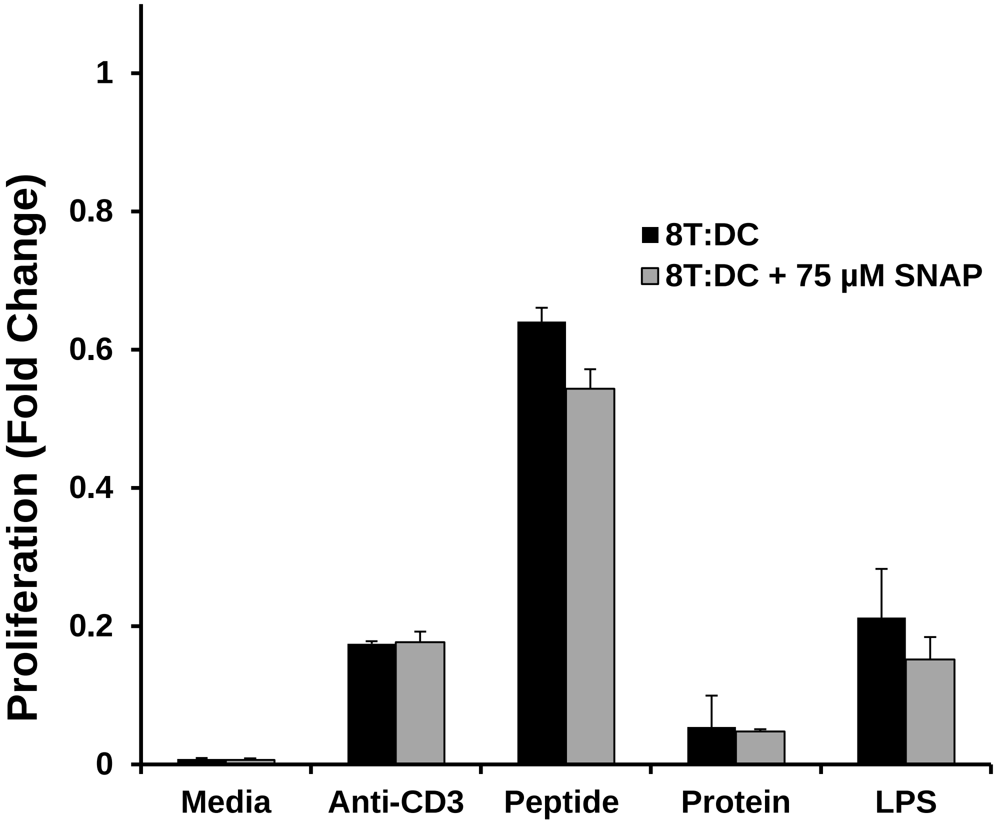

Supplemental Figure 2. The effects of NO on DC function were evaluated using transgenic mice that express a T cell receptor (TCR) specific for a defined antigen, namely ovalbumin (OVA). DCs and $CD4^+$ T cells purified from the spleen and lymph nodes of an OT-II mouse were treated with anti-CD3 (global T cell activating treatment), the OVA 329-337 peptide, or whole OVA protein in the presence or absence of a NO donor SNAP. Lipopolysaccharide (LPS) is a control for nonspecific inflammation. When the ratio of T cells to DCs was increased to 8:1 after addition of 75 µM SNAP, the level of antigen presentation decreased but there still appeared to be a NO dependent inhibition of antigen presentation from DCs to $CD4^+$ T cells (15.2 ± 5.7 % decrease). A fold change of 1 on this graph is equivalent to a ratio of 4T:DC.

Supplemental Figure 3

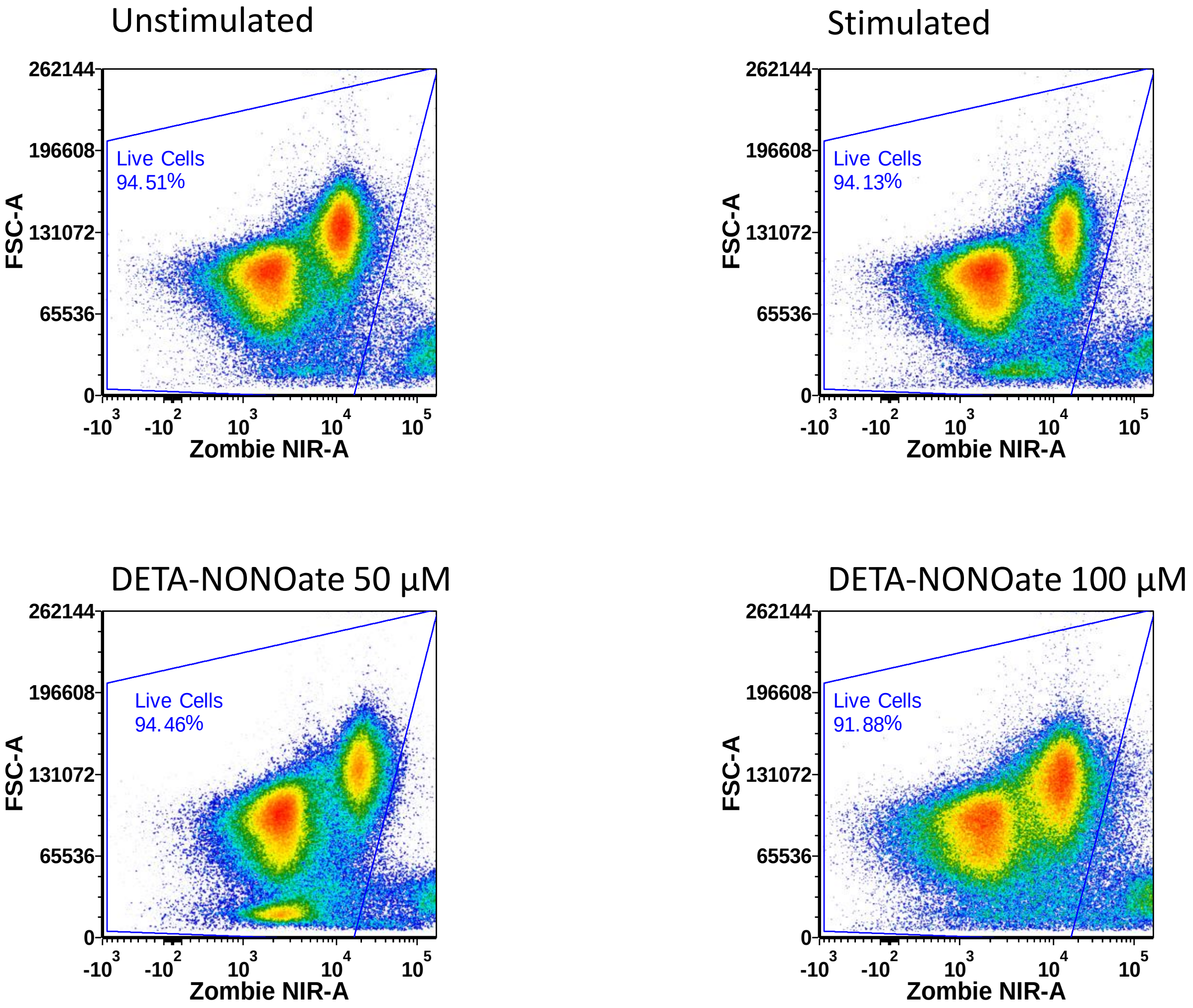

Supplemental Figure 3. The ability of T cells derived from the OTII mouse to detect OVA specific tetramers was measured in the presence and absence of the nitric oxide donor, DETA-NONOate. Specifically T cells were stimulated with 10 µg/mL OTII peptide cultured for 48 hours. After 48 hours OTII specific tetramers were measured on $CD3^{+}CD4^{+}$ T cells that were unstimulated, stimulated for 48 hours without DETA-NONOate, stimulated for 48 hours with 50 µM DETA-NONOate, and stimulated for 48 hours with 100 µM DETA-NONOate (Fig. 3C-F). Each experiment was performed in duplicate utilizing cells from different OTII mice and demonstrated similar results. A representative plot is presented for each condition. The decrease in recognition of OT II tetramers was 68.3 ± 1.1% and 76.5 ± 1.3% at 50 and 100 µM DETA-NONOate, respectively (Fig 3C-3F). The viability of the cells was not significantly altered as demonstrated by this figure.

Supplemental Figure 4

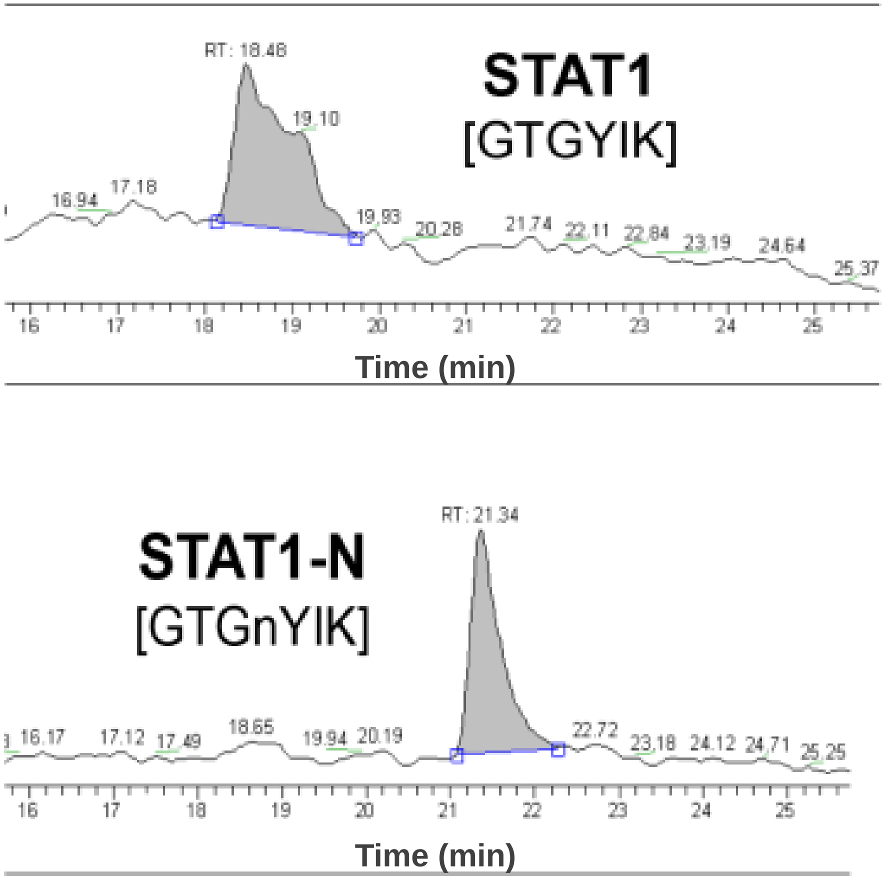

Supplementary Figure 4. Chromatogram for the STAT1 peptide and the nSTAT1 peptide. Notice the shift in the retention time on the column for the nSTAT1 peptide.

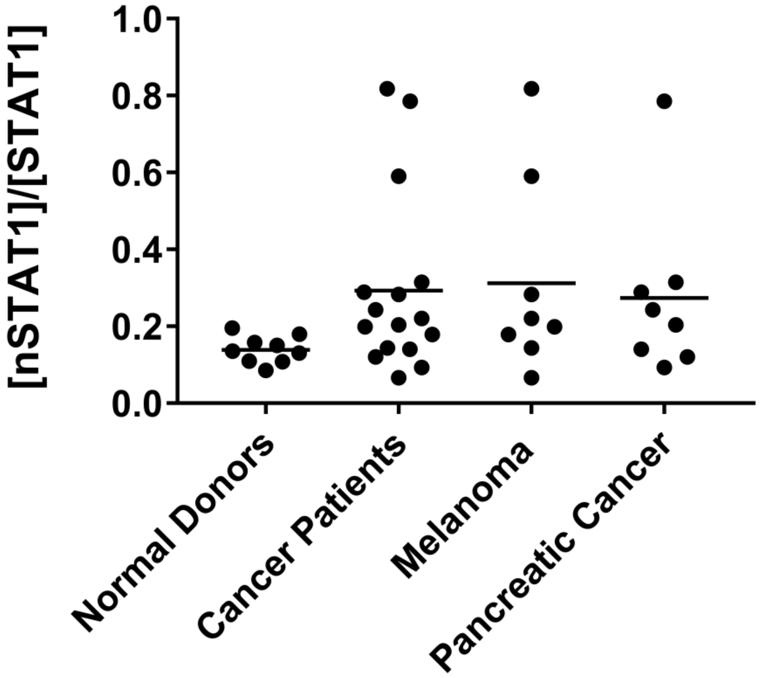

Supplemental Figure 5: [nSTAT1]/[STAT1] ratios for each cohort. The cohorts are presented for illustration purposes only.

	% MDSC	% MDSC (DAF-FM$^+$)	MDSC (DAF-FM$^+$) Geometric mean
**Normal**	0.19	85.7	56840
**Melanoma**	6.44	99.9	125274
**Pancreatic**	15.80	99.8	88808

Supplemental Table 1. Normal PBMC have few cells with the MDSC phenotype and reduced levels of NO. Normal PBMC derived from the peripheral blood of a normal donor were gated for HLADR^{neg}CD11b$^+$CD33$^+$ cells and these same cells were stained for NO using DAF-FM. PBMC derived from the peripheral blood of a melanoma or pancreatic cancer patients were also stained for NO in the MDSC phenotype. As seen elsewhere in the literature, there are few cells with the MDSC phenotype in the normal donor, and these cells have less NO.

Supplemental Table 2

	[STAT1] (nM)	[nSTAT1] [nM)	[nSTAT1]/STAT1]
Sample 1 (no SNAP)	19.5	2.97	
Sample 1 (SNAP)	16.0	4.83	1.98
Sample 2 (no SNAP)	26.103	4.781	
Sample 2 (SNAP)	34.153	17.089	2.77
Sample 3 (no SNAP)	27.058	3.678	
Sample 3 (SNAP)	82.323	16.724	1.43

Supplemental Table 2. The concentrations of [STAT1] and [nSTAT1] for three independent PBMC samples treated with the nitric oxide donor SNAP overnight are displayed to demonstrate how we calculated the [nSTAT1]/[STAT1] ratio.
